# Investigation of Delayed Transfusion Reactions in Sickle Cell Disease Patients Polytransfused in the Brazilian Amazon

**DOI:** 10.3390/hematolrep16030049

**Published:** 2024-08-01

**Authors:** Lorena Alves Santos, Anne Cristine Gomes de Almeida, Andrea Monteiro Tarragô, Nina Rosa Gonçalves da Silva, Juliana Nascimento Vitoriano da Silva, Mônica Moura de Souza, Monik Oney Oliveira Nascimento, Marcelo Reis do Nascimento, Ana Caroline dos Santos Castro, Cinthia Xerez de Albuquerque, Evilázio Cunha Cardoso, José Pereira Moura Neto, Sérgio Roberto Lopes Albuquerque

**Affiliations:** 1 Programa de Pós-Graduação em Ciências Aplicadas à Hematologia da Universidade do Estado do Amazonas (PPGH-UEA), Manaus 69050-001, AM, Brazil; lorenaalvessantos7@gmail.com (L.A.S.); anne.almeida.gb@gmail.com (A.C.G.d.A.); andrea_s_monteiro@hotmail.com (A.M.T.); juliananvdasilva@gmail.com (J.N.V.d.S.); monicamoura728@gmail.com (M.M.d.S.); jpmnneto@gmail.com (J.P.M.N.); 2 Programa de Pós-Graduação em Imunologia Básica e Aplicada da Universidade Federal do Amazonas (PPGIBA-UFAM), Manaus 69067-005, AM, Brazil; monikoney@gmail.com (M.O.O.N.); marcelo.reis.reis@gmail.com (M.R.d.N.); castroanac@hotmail.com (A.C.d.S.C.); 3 Fundação Hospitalar de Hematologia e Hemoterapia do Amazonas (HEMOAM), Manaus 69050-001, AM, Brazil; ninarosagoncalves@gmail.com (N.R.G.d.S.); diretoriaclinica@hemoam.am.gov.br (C.X.d.A.); evilazio.cardoso@hemoam.am.gov.br (E.C.C.); 4 Universidade Federal de Juiz de Fora—Campus Governador Valadares, Governador Valadares 35032-620, MG, Brazil

**Keywords:** delayed transfusion reactions, sickle cell disease, alloantibody, hemolysis

## Abstract

Background: Sickle cell disease (SCD) affects approximately 100,000 people in the United States and millions worldwide, with the highest prevalence of 70% of SCD being found in individuals of African ethnicity. Delayed hemolytic, alloimmunization, and anamnestic transfusion reactions in multiple transfusion patients need to be investigated and managed to avoid a worsening of the patient’s clinical status. Objective: This paper aims to investigate delayed transfusion reactions in SCD patients who were polytransfused in the Brazilian Amazon. Material and Methods: The clinical and laboratory indicators of SCD patients with more than four transfusions were investigated. The patients were treated at the Fundação Hospitalar de Hematologia e Hemoterapia do Estado do Amazonas, Brazil. Results: A total of 44 polytransfused patients with SCD were followed. Regarding Rh phenotype, it was possible to observe a frequency of 26.6% (12) patients with the RZRZ (DCE/DCE) phenotype, in addition to 4.5% (two) patients with *RH* and *RHCE* variants. It was also possible to observe 20.5% (nine) patients with an alloimmunization reaction, who presented the following alloantibodies: anti-RhD, anti-E, anti-K, anti-Jk^b^, anti-N, anti-S, and anti-Di^a^, two of which are unidentified. Of these, four (44.4%) patients also presented autoantibodies, anti-e, and three unidentified antibodies, and four (44.4%) patients presented an anamnestic reaction, with anti-RhD, K, and Jkb antibodies. Of the 44 patients monitored, 54.4% (24) had clinical and laboratory indicators of a delayed hemolytic reaction. Conclusion: Delayed transfusion reactions, often neglected, occur frequently. Therefore, transfusions need to be monitored for at least 28 days, with medical investigation of clinical and laboratory indicators to make greater use of this therapeutic resource.

## 1. Introduction

Sickle cell disease (SCD) affects approximately 100,000 people in the United States and millions worldwide, with the highest prevalence of 70% of SCD being found in individuals of African ethnicity [1,2]. In Brazil, prevalence is discussed as a public health problem due to the prevalence of 2% to 8% in the population, and it is estimated that an average of 3500 children are born with SCD [3,4]. It is an autosomal recessive genetic disease caused by homozygosity of the beta-S allele (b^s^), located on chromosome 11p15.5, which differs from the wild-type B allele by a single missense polymorphism, dbSNP rs334 (T; T), in which GTG is replaced by GAG in the sixth codon of the beta-globin gene. This leads to the replacement of the amino acid glutamic acid (Glu) by Valine (Val) in the sixth polypeptide chain, and the expression of mutated hemoglobin (Hb) HbS (alpha 2 beta S 2) in the red blood cells of individuals with SCD [2,5].

The pathophysiology of SCD is characterized by HbS polymerization, vessel occlusion, and hemolysis-mediated endothelial dysfunction, in addition to inflammation that leads to clinical disease, hemolytic anemia, and microvascular vaso-occlusion cycles leading to ischemia, target organ reperfusion, and infarction [6]. Individuals with symptomatic SCD have chronic transfusion as a therapeutic measure, aiming at the replacement of erythrocyte concentration, hemoglobin, and blood volume. However, late reactions, such as alloimmunization, shorten the survival of transfused red blood cells due to the presence of alloantibodies, which adsorb around the red blood cells, destroying the cell membrane and causing a delayed hemolytic reaction (DHR). It is estimated that 1.6% to 11% of transfused patients with SCD develop overt transfusion reactions with increased fatigue, jaundice, dark urine, fever, and/or pain [7,8].

In Brazil, great racial miscegenation constitutes a major challenge for transfusion services regarding the availability of red blood cell concentrates with compatible erythrocyte antigens for multiple transfusion patients, such as in the case of sickle cell anemia. The risk of hemolytic transfusion reactions secondary to alloantibodies and the risk of iron overload and hyperhemolysis secondary to red blood cell transfusion are also weighed against the benefits of transfusion. Hyperhemolysis refers to the development of severe anemia in which the hemoglobin level after transfusion is lower than before, which may manifest immediately or later, being associated with a new alloantibody or a previous antibody that was not detected in the antibody screening. However, it may not be associated with an alloantibody [9,10].

The development of an alloantibody after transfusion may result in an alloimmunization reaction or a DHR. Epidemiological data from the American Blood Bank Association point to a risk of up to 70% of alloimmunization for blood group systems such as Rh, Kell, Kidd, Duffy, and MNS for patients with hemoglobinopathies, including SCD [9]. Fever and anemia occurring days to weeks after transfusion of a red blood cell component characterize a delayed transfusion reaction, and the associated hemolysis is more prolonged than an acute hemolytic reaction and typically does not have the characteristic signs and symptoms, although some patients may develop jaundice and leukocytosis [11]. In these cases, transfusion of phenotyped red blood cell concentrate to the main blood group systems Rh, Kell, Kidd, Duffy, and MNS is indicated, with the aim of preventing the alloimmunization reaction in polytransfused patients and facilitating the selection of blood components for those previously alloimmunized [9,12].

In the State of Amazonas, approximately 300 people live with SCD, and diagnosis and follow-up are provided by the Hospital Foundation of Hematology and Hemotherapy of Amazonas (HEMOAM), a reference unit for the diagnosis and treatment of blood diseases [13]. Therefore, it is important to investigate the frequency of delayed alloimmunization and hemolysis reactions in patients with polytransfused SCD. The objective of this study was to characterize the immunohematological profile of DHR in patients with SCD who were polytransfused at HEMOAM.

## 2. Materials and Methods

### 2.1. Local Study

This study was carried out at the HEMOAM, which is a reference center for multidisciplinary follow-up of patients with sickle cell anemia, located in the city of Manaus, Amazonas, Brazil.

### 2.2. Ethical Aspects

This study was approved by the HEMOAM Research Ethics Committee through document 5.221.858 and Certificate of Presentation of Ethical Review 52069321.8.0000.0009, according to resolution 466/2012 of the National Health Council, which advocates the guidelines and Regulatory Norms for Research Involving Human Beings, including evidence that the research was conducted ethically in accordance with the World Medical Association’s Declaration of Helsinki. All participants signed the study consent form.

### 2.3. Study Participants

The participants were patients diagnosed with SCD expressing Hb SS with chronic transfusion of red blood cells (RBCs) as a therapeutic protocol, consisting of traditional and special blood components, as well as conjugated blood components such as filtered and irradiated red blood cell concentrate.

Chronic transfusion in study patients was indicated in cases of patient return after transfusion and verification of HB reduction to pre-transfusion levels, reticulopenia, an increase in spherocytes, a positive Direct Antiglobulin test, a reduction in haptoglobin, an increase in dehydrogenase lactic acid, an irregular positive antibody test, severe vaso-occlusive crises, and symptomatic anemia.

### 2.4. Inclusion and Exclusion Criteria

The inclusion criteria were patients with SCD of both sexes, who had received three or more units of RBC in a twelve-month period, and for whom data on the history of transfusions, previous and/or updated immunohematological, hematological, and biochemical data were available. Patients who did not comply with the transfusion schedule and those with incomplete records were excluded.

### 2.5. Participant Follow-Up

The initial approach to the patient was made at the time of waiting for the transfusion or at the time of the collection of pre-transfusion tests. After signing the informed consent form, a questionnaire was given to the participants with questions about sociodemographic data, transfusion history, and occurrence of previous transfusion reactions, which were later confirmed through the participants’ medical records. The patient’s identification number and the type of blood component received were also recorded.

Transfusions of a hemocomponent are completed within 4 h of the beginning of the transfusion, with a duration of 1–2 mL/min (60–120 mL/h). Patients with confirmed DHR status received a transfusion of 2 red blood cell concentrates within 24 h, according to the HEMOAM Transfusion Guide. If there are no clinical complaints 30 min after transfusion and if there are no clinical manifestations of transfusion reactions in subsequent routine visits, a new transfusion procedure will be scheduled for the participant. If there is an intercurrence reported by the patient, a medical investigation will be carried out by the blood center regarding the probable late transfusion reaction. Before the transfusion procedure, a new interview was conducted to verify the presence of complications reported by the patients, and when necessary, a new sample was collected for the comparison of pre- and post-transfusion parameters.

### 2.6. Phenotyping Procedures

Erythrocyte phenotyping: This was performed using the gel centrifugation technique (BioRad, Hercules, CA, USA), using antibodies to the main antigens of the Rh (D, C, c, E, e), Kell (K, k), Kidd (Jk^a^, Jk^b^), Duffy (Fy^a^, Fy^b^), and MNS (M, N, S, s) systems, as well as a sample of erythrocytes from the patient and donor, which was suspended in saline medium.

Detection and identification of unexpected antibodies: This was performed using plasma and screening red blood cells (BioRad) suspended in saline medium and incubated at different temperatures of 4 °C, Room Temperature (RT) (20–25 °C), and 37 °C, followed by an antiglobulin test. Tests at 4 °C and RT seek to detect all unexpected antibodies that act below 37 °C, which can cause a transfusion reaction in titers above 64. The 37 °C antiglobulin test seeks to detect potentially hemolytic antibodies. The identification of detected alloantibodies was performed by testing the patient’s plasma with a panel of 10 (BioRad) and 16 different vials of phenotyped red blood cells (Fresenius, Bad Homburg, Germany). When an antibody was detected, successive dilutions of the patient’s plasma were performed. The titers ranged from 1:2 to values greater than 1:1024, through the reading of the reaction intensity in crosses, which also allows the definition of scores that could differentiate concentrations of two alloantibodies with the same titer. Plasma treatment with Dithitreitol (DTT) was also performed to verify the class of the detected antibody, and an autocontrol test was performed to detect autoantibodies in addition to the alloantibody found. A direct antiglobulin test (DAT) was performed to detect the presence of antibodies and/or proteins of the complement system linked to the individual’s erythrocyte membrane, which may be allo- or autoantibodies originating from various immunological reaction processes. Cross-matching was performed using the indirect antiglobulin test (IAT) at 37 °C, using the patient’s plasma and the donor’s erythrocytes in order to detect potentially hemolytic antibodies.

Transfusional reactions definitions: An anamnesthetic response may be characterized by a negative IAT result on pretransfusion testing of the current transfusion; detection of alloantibodies within 30 days of the current transfusion; and observation of the presence of the same alloantibody detected in pre-transfusion tests from previous transfusions (confirmed by data recorded in Hemosys). Primary alloimmunization can be characterized by a negative IAT result in the pre-transfusion tests of the current transfusion; detection of alloantibodies within 30 days after the current transfusion; and observation of a negative IAT result in all pre-transfusion tests of previous transfusions (confirmed by the data recorded in Hemosys). For the analysis of the post-transfusion hemoglobin tests, the patients tested were distributed into the Confirmed, Possible, Probable, and Discarded DHR groups. Confirmed *DHR* occurs when a new antibody is identified between 24 h and 28 days after the transfusion and the transfusion carried out at the institution itself is the only possible cause for the appearance of irregular antibodies. Probable *DHR* occurs when the investigation has already been completed, or is still ongoing, presenting evidence (clinical/laboratory/temporal links) that indicates an association with transfusion; however, there are other causes that can explain the signs and symptoms. Possible *DHR* occurs when the investigation has already been completed, or is still ongoing, presenting evidence (clinical/laboratory/temporal links) that indicates the association of signs and symptoms to other causes; however, the association with the transfusion cannot be discarded. Discarded *DHR* occurs when the already completed investigation presents evidence (clinical/laboratory/temporal links) that clearly demonstrates the association of the adverse event to another cause(s) and not to the transfusion.

Compatibility Percentage Test: Compatibility was calculated through a test developed and validated for this study, which involved the offer of a bag with the same negative (compatible) antigens that the patient had, i.e., Rh (D, C, c, E, e); Kell (K, k); Kidd (Jk^a^, Jk^b^); Duffy (Fy^a^, Fy^b^); and MNS (M, N, S, s), comprising a total of 15 antigens. This test evaluates the percentage chance of the patient developing a delayed reaction to immunization from the moment he received any of these antigens that did not have them in the phenotyping. To calculate the percentage, the following formula was used: percentage of compatibility (%) = (number of negative antigens in the bag * 100)/number of negative antigens in the patient.

### 2.7. Data Analysis

Laboratory data and the clinical characteristics of the individuals in the study were recorded in spreadsheets prepared with Microsoft Excel 2020 (Microsoft Corporation) software. Statistical analysis was performed using the GraphPad Prism software (v.5.0). Numerical variables were expressed as mean ± standard deviation or median (confidence interval) due to the absence of normal distribution. Categorical variables were expressed as absolute value (n) and relative frequency (%). Statistical analysis between independent groups, qualitative variables, and continuous quantitative variables was performed using descriptive statistics.

## 3. Results

The immunohematological profiles of 44 patients, who were part of the transfusion flow scheduled at the blood center, were investigated. The characteristics of the patients are described in Table 1.

### 3.1. Frequency of Rh, Kell, Kidd, Duffy, and MNS Phenotypes

All patients were phenotyped for the Rh and Kell systems, but only 81.8% were phenotyped for the Kidd system, 86.8% for the Duffy system, and 90.9% for the MNS system. Regarding the Rh phenotype, it was possible to observe a frequency of 26.6% (12) with the RZRZ phenotype (DCE/DCE), in addition to 4.5% (two) patients with RH and RHCE variants, respectively. The first positive RhD presented partial anti-RhD, and the second with a mutation in *RHCE* (RHCE* Ce/RHCE* ce (733G) presented auto-anti-e in addition to anti-E and anti-Di^a^. A frequency of 4.5% was also observed with the K + k − phenotype. These data are described in Table 2.

### 3.2. Percentage Compatibility between the Blood Component and Recipient Regarding the Rh, Kell, Kidd, Duffy, and MNS Systems

**Figure 1:**
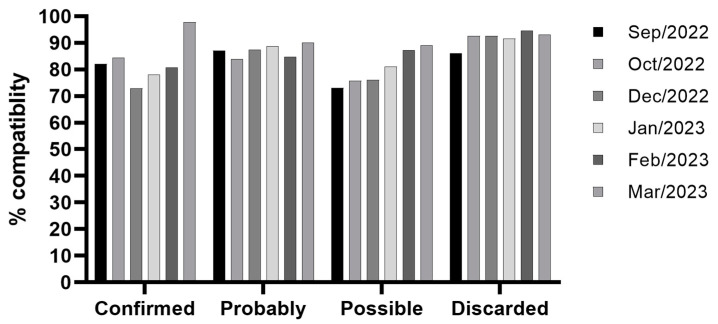
Percentage of compatibility between the blood component and patient in the months evaluated in this study (*p* > 0.05). The compatibility percentages are separated into confirmed, probable, possible, and discarded DHR.

It was observed that most of the patients in the study had 80–90% compatibility with the Rh, Kell, Kidd, and MNS systems. The compatibility percentages are shown in Figure 1, separated according to the clinical outcome of confirmed, probable, possible, and discarded DHR. Statistical analysis was performed on a single group of sickle cell patients, comparing their phenotyping with the phenotyping of donors selected for transfusion. 

### 3.3. Frequency of Delayed Anamnestic and Primary Alloimmunization Reactions

After a retrospective search for the presence of alloantibodies and autoantibodies in the patients through registration in the data system, the cases of primary alloimmunization and anamnestic reactions were defined based on pre-transfusion tests. It was observed that 20.5% (9/44) of the patients had a delayed alloimmunization reaction, and it was possible to determine the antibodies involved, except in the case of patients ID-60 and ID-78 (Table 3), whose specificities were not identified using routine red blood cell panel kits. Four of these nine patients (44.4%) also had autoantibodies confirmed by positive autocontrol test results, in addition to the identified alloantibodies. It was also found that four of these nine patients (44.4%) presented an anamnestic reaction. The following patients stand out: ID-74 had an *RHD* variant, which was not molecularly identified, presenting partial anti-RhD allo with anti-RhD. Patient ID-81 had a *RHCE* with RHCE* Ce/RHCE* ce genotype (733G) variant, presenting auto-anti-e, anti-E, and anti-Di^a^ alloantibodies. The immunohematological profiles of the patients are described in Table 3.

### 3.4. Delayed Hemolytic Reactions

The parameters applied for the diagnosis of DHR were based on the criteria defined in the Brazilian Hemovigilance Manual [14] and by Gerritsma et al., 2021 [15]. The criteria to be considered were a delayed hemolytic reaction and a reduction in hemoglobin and/or haptoglobin levels, an increase in spherocytes and/or reticulocytes, a positive direct antiglobulin test, and an increase in LDH level. The criterion to characterize alloimmunization was the presence of an alloantibody determined by the cold, RT, and hot antibody tests.

For the analysis of the post-transfusion hemoglobin tests, the patients tested were distributed into the Confirmed, Possible, Probable, and Discarded groups, and analyzed individually to verify whether there was a DHR, as shown in Table 4. The transfused blood component did not result in the expected increase in Hb, <1 g/dL, compared to the pre-transfusion test in 24 patients (54.5%) distributed among the groups analyzed. Regarding the post-transfusion LDH (lactate dehydrogenase) test, 14 patients (31.8%) tested had values above the reference value (125.0 U/L and 200.0 U/L). These results were considered indicative of possible delayed hemolytic reactions. However, laboratory indicators were analyzed together with the clinical symptoms of a hemolytic reaction, mainly pain, fever, weakness, and jaundice, which made it possible to rule out evidence of a late reaction in 45.6% of patients monitored.

All patients with confirmed DHRs did not show an increase in hemoglobin of at least 1 g/dL after the transfusion of one red blood cell concentrate, and LDH values were greater than 480 U/L, in addition to the frequency of transfusion being on average 20 days/month, with hemoglobin level less than 7 g/dL. The symptoms presented were malaise, jaundice, and headache. We highlight the case of the *RHCE* variant patient, with auto anti-e and later alloimmunized with anti-E and anti-Di^a^, who presented intense pallor, dizziness, and headache, in addition to two episodes of dark urine after 14 days after transfusion. Table 5 presents the results found in the 44 patients monitored.

## 4. Discussion

This study is the first to describe the immunohematological profile of delayed transfusion reactions in patients with SCD who received multiple transfusions at HEMOAM. It is important to note that all patients with sickle cell anemia treated at HEMOAM are phenotyped before the first transfusion for the Rh, Kell, Kidd, Duffy, and MNS systems. It turns out that we do not always have a donor phenotype available that is 100% identical to that of the patient, in addition to the need for extremely urgent transfusions.

In all 44 patients, Rh and Kell phenotyping was respected in the transfusions performed; however, three cases of alloimmunization with Rh system antibodies were observed, one being partial anti-RhD in a *RH* variant patient, one being anti-E in a *RHCE* variant patient with anti-e, and an anti-E in an anamnestic response in a patient who was urgently transfused in a city emergency room that did not have RBC phenotyped for transfusion compatible with the Rh system. An anti-K alloimmunization was also observed in an anamnestic response with the same situation mentioned above. Interestingly, Figure 1 demonstrates that there was no statistical significance (*p* > 0.05) between the confirmation of a delayed transfusion reaction and the percentage of compatibility, especially for antigens from the Kidd, Duffy, and MNS systems, clearly demonstrating their immunogenic capacity. However, we also observed two alloantibodies and three autoantibodies that could not be identified, and their specificities may be directed to antigens that were not within the scope of our phenotype panels or drug-induced antibodies.

The immunohematological characterization of SCD patients who presented delayed reactions can contribute to better pre-transfusion management, suggesting, for example, the profile of antigens that must be respected when using blood, when necessary. In this survey, it was possible to verify the complexity of the phenotypic frequency of the Rh system in this group of patients in the State of Amazonas, revealing that 26.6% of individuals have the RZRZ phenotype (DCE/DCE). It is known that there is great difficulty in performing compatible transfusions in the Rh system in these patients, since the state of Amazonas has a high frequency of blood donors with positive ‘e’ antigens (94.53%) and positive ‘c’ antigens (79.1%), according to the HEMOAM data center. In another study carried out in Brazil, it was found that among polytransfused sickle cell patients, the most frequent phenotype was R1r and R0R (41.0 and 20.8%, respectively), with 0% of RZRZ individuals [16], suggesting that this profile may be characteristic of sickle cell patients from the Brazilian Amazon.

Furthermore, two patients (4.5%) were found with variants of *RHD* and *RHCE*, the first of which had partial anti-RhD, proven through the presence of anti-RhD in his plasma, although it has not been molecularly characterized to date. The second was identified with the variant RHCE* Ce/RhCE* ce genotype (733G), presenting auto-anti-e and anti-E and anti-Di^a^ alloantibodies. The second case demonstrates a special difficulty for transfusion since the patient presented anti-e and anti-E, with physicians recommending the transfusion of R1R1 red blood cells, opting for incompatibility with auto-anti-e and compatibility with the anti-E alloantibody and observing effective medical management as it was possible to observe a better use of the transfused red blood cells. Fasano et al. (2019), in a cohort study with 403 sickle cell patients, found RH1(D), 20.8% RH2(C), and 3.5% RH5(e), with the following respective frequencies of alloimmunization: 17.6% RhD variants, 14.3% C-variants, and 7.1% e-variants [17]. In Ghana, research on 154 sickle cell patients aiming to find *RHD* and *RHCE* variants obtained results from three patients with multiple antibodies against Rh system antigens, such as anti-RhD, partial anti-RhD, anti-C, partial anti-C, and auto anti-e; these patients had positive RhD with *RHD* and *RHCE* deletion genotyping [18].

In this study, the frequency of alloimmunization was 20.4% in the patients studied, with anti-E, anti-RhD variant, anti-K, anti-Jk^b^, anti-N, anti-S, and anti-Di^a^ antibodies. In the study by Silvy et al. (2014), 33.5% of alloimmunization against the Rh system was described in 198 polytransfused patients [19]. Boateng et al. (2019)’s study, conducted with 150 patients with SCD from Ghana, found a 25% frequency of alloimmunization of anti-RhD, anti-C, anti-E, and partial antibodies, detected through serological tests and confirmed through genotyping tests [18]. In a study carried out in São Paulo, the presence of alloantibodies was detected in 22.6% of phenotyped sickle cell patients (N = 53), with Anti-K, Anti-C, and Anti-Di^a^ being the most common antibodies [16]. In another study that evaluated alloimmunization in sickle cell patients in a hospital in the Federal District, the frequency of alloimmunization was 36.4% (28/77), with the main alloantibodies being reported against Rh (“C” and “E”) antigens and Kell antigens [20]. In a cohort study performed with pediatric patients in sub-Saharan Africa, 16% presented positive irregular antibodies directed against the Rhesus (30.76%) and Kell (69.24%) blood group systems [21]

In this study, 4/9 patients (44.4%) had anamnestic responses, i.e., they had previously identified alloantibody records, but these were not detected during pre-transfusion tests due to a drop in titration as the transfusions of packed red blood cells were negative for the antigen for which the patient had the antibody. These patients presented the previously recorded antibody again after transfusion within the follow-up of this study, characterizing an anamnestic response (ID-31 with anti-K, ID-74 with anti-RhD, ID-91 with anti-JK^b^, and ID-93 with anti-E). This happened due to an emergency transfusion outside of the coordinating blood center, in emergency services in the capital, demonstrating the need to implement a computerized system with information on previous transfusions to be verified in all transfusion services in the city with mandatory consultation of the patient’s history, i.e., whether they have an alloantibody and whether they have already had transfusion reactions so that a special, phenotyped, or even irradiated or filtered blood component can be provided. A total of 4/9 patients (44.4%) had their autoantibodies detected in addition to the identified allo antibodies, but three were not identified.

Fasano et al. (2019) confirmed this type of anamnestic reaction, describing that throughout some transfusions with negative antigens, this alloantibody remains without stimulation, reducing the titer to thresholds below the sensitivity of the antiglobulin reagent; thus, the unexpected antibody becomes undetectable [17]. Andrade et al. (2022), in a study with 3178 patients treated at a blood center in Northeast Brazil, also described the presence of anti-Jkb (5%), anti-S (14%), and anti-Dia (29%) in anamnestic responses [22].

Regarding the occurrence of delayed hemolytic reactions, in addition to immunohematological evidence, laboratory parameters were also evaluated. A frequency of 13.6% of DHR was identified in sickle cell patients, with a low increase in Hb in 54.5% of patients and high rates of LDH results in 31.8%, showing signs of extravascular hemolysis. These findings corroborate the results found by Gerritsma et al. (2021), who followed 664 patients with SCD in the Netherlands and found that 60% of patients fulfilled at least one of the criteria for DHR (Hb increase < 1.5 g/dL post-transfusion or Hb less than pre-transfusion, dark urine, altered LDH results, and recurrence of vaso-occlusive crises) during transfusion follow-up [15].

One of the patients who had DHR and was followed up in the study had variant *RHCE*, alloimmunization with auto anti-e and, later, anti-E and anti-Di^a^, presenting intense pallor, dizziness, headache, and two episodes of dark urine 14 days after transfusion. A study conducted by Pirenne et al. (2018) described similar symptoms in DHR, such as fever, dizziness, vomiting, headache, jaundice of the skin and mucous membrane, and in severe cases, dark urine and convulsion [23].

## 5. Conclusions

Delayed transfusion reactions, often neglected, occur frequently. Therefore, transfusions need to be monitored for at least 28 days, with medical investigation of clinical and laboratory indicators to make greater use of this therapeutic resource. To reduce the occurrence of delayed reactions, it is recommended to limit the number of transfusions and, when there is a need for transfusion, only use blood that has undergone extended phenotyping.

## Figures and Tables

**Table 1:** Baseline characteristics of patients.

Characteristics	N = 44
Age in years, median (95% CI)	18.5 (2–72)
Age in years, n (%)	
2–11	13 (29.5)
12–17	13 (29.5)
18–39	11 (25)
40–59	5 (11.3)
>60	2 (4.5)
ABO Blood Group, n (%)	
A	14 (31.8)
B	1 (2.2)
AB	2 (4.5)
O	27 (61.3)
Transfusions performed, mean (min–max)	16.68 (3–37)
Alloimmunization reactions (min–max)	10 (1–4)

**Table 2:** Phenotypic frequency of Rh, Kell, Kidd, Duffy, and MNS systems in polytransfused SCD patients.

Blood Group Systems	n (%)
RH	44 (100)
R1R1 (DCe/DCe)	11 (24.4)
R1R2 (DCe/DcE)	10 (22.2)
R2R2 (DcE/DcE)	2 (4.4)
R2r (DcE/dce)	1 (2.2)
R0r (Dce/dce)	4 (8.8)
RZRZ (DCE/DCE)	12 (26.6)
rr (dce/dce)	3 (6.6)
RHCE* Ce/RHCE* ce (733G)	1 (2.2)
Kell	44 (100)
K-k+	27 (61.3)
K+k-	2 (4.5)
K+k+	2 (4.5)
Kp (a-b+)	13 (29.5)
Kidd	36 (81.8)
Jk(a+b+)	17 (37.7)
Jk(a+b-)	11 (25)
Jk(a-b+)	8 (17.7)
Duffy	38 (86.4)
Fy(a+b-)	11 (25)
Fy(a+b+)	15 (34)
Fy(a-b+)	11 (25)
Fy(a-b-)	1 (2.2)
MNS	40 (90.9)
M+N+S-s+	5 (11.3)
M+N+S+s+	7 (15.9)
M-N+S-s+	1 (2.2)
M+N-S+s+	14 (31.8)
M+N-S-s+	2 (4.5)
M-N+S+s+	4 (9.0)
M+N-S+S-	4 (9.0)
M+N+S+s-	2 (4.5)
M-N-S+s+	1 (2.2)

**Table 3:** Immunohematological profiles of patients with SCD on polytransfusions.

ID	Allo Immunization							Anamnestic Reaction
	Allo Antibody	Title	Class	PAI 37 °C	PAI 4 °C	Autocontrol	Title	
31	Anti-K	1:16	IgG	yes	no	No	-	yes
60	N.I. *	1:8		no	yes	No	-	no
63	Anti-N	1:8	IgM	yes	yes	No	-	no
74	Anti-RhD **	1:128	IgG	yes	yes	No	-	yes
78	N.I. *	1:8		no	yes	No	-	no
81	Anti-E; Anti-Di^a^	1:64	IgG/IgM	yes	yes	yes auto anti-e	1:64	no
91	Anti-Jk^b^	1:16	IgG	yes	yes	yes N.I *	1:16	yes
92	Anti-S	1:16	IgG	no	yes	yes N.I *	1:8	no
93	Anti-E	1:64	IgG	yes	no	yes N.I *	1:16	yes

* Not Identified, ** Partial.

**Table 4:** Average post-transfusion hemoglobin increase.

	Confirmed	Probable	Possible	Discarded
n	6	9	9	13
Hb pre-transfusion (g/dL)	5.9 ± 1.2	6.8 ± 2.1	5.9 ± 1.3	6.7 ± 1.3
Hb post-transfusion (g/dL)	6.35 ± 1.2	7 ± 1.4	6.3 ± 0.9	8.4 ± 1.1

**Table 5:** Delayed Reaction frequencies in patients monitored.

Delayed Reaction	n	%
**Alloimmunization**		
Allo antibodies	9/44	20.5
Anti-RhD	1	2.3
Anti-K	1	2.3
Anti-Jk^b^	1	2.3
Anti-E	2	4.5
Anti-Di^a^	1	2.3
Anti-N	1	2.3
N.I.	2	4.5
Allo + Auto antibodies	4/9	44.4
Auto anti-e	1	11.1
N.I.	3	33.3
Anamnestic	4/9	44.4
Anti-RhD	1	11.1
Anti-E	1	11.1
Anti-K	1	11.1
Anti-Jk^b^	1	11.1
**Hemolytic**		
Confirmed	6/44	13.6
Probable	9/44	20.4
Possible	9/44	20.4
Discarded	24/44	45.6

N.I. = Not Identified.

## Data Availability

All our data are deposited in a public bank, in which my research group owns. Can make it available whenever requested upon application to researcher coordinator email: sergiorlalbuquerque@gmail.com.
